# Singing classes for chronic obstructive pulmonary disease: a randomized controlled trial

**DOI:** 10.1186/1471-2466-12-69

**Published:** 2012-11-13

**Authors:** Victoria M Lord, Victoria J Hume, Julia L Kelly, Phoene Cave, Judith Silver, Maya Waldman, Chris White, Cayley Smith, Rebecca Tanner, Melissa Sanchez, William D-C Man, Michael I Polkey, Nicholas S Hopkinson

**Affiliations:** 1 NIHR Respiratory Biomedical Research Unit at Royal Brompton and Harefield NHS Foundation Trust and Imperial College, Sydney Street, London, SW3 6NP, UK

**Keywords:** COPD, Singing, Qualitative, Randomised controlled trial, Rehabilitation

## Abstract

**Background:**

There is some evidence that singing lessons may be of benefit to patients with chronic obstructive pulmonary disease (COPD). It is not clear how much of this benefit is specific to singing and how much relates to the classes being a group activity that addresses social isolation.

**Methods:**

Patients were randomised to either singing classes or a film club for eight weeks. Response was assessed quantitatively through health status questionnaires, measures of breathing control, exercise capacity and physical activity and qualitatively, through structured interviews with a clinical psychologist.

**Results:**

The singing group (n=13 mean(SD) FEV_1_ 44.4(14.4)% predicted) and film group (n=11 FEV_1_ 63.5(25.5)%predicted) did not differ significantly at baseline. There was a significant difference between the response of the physical component score of the SF-36, favouring the singing group +12.9(19.0) vs -0.25(11.9) (p=0.02), but no difference in response of the mental component score of the SF-36, breathing control measures, exercise capacity or daily physical activity. In the qualitative element, positive effects on physical well-being were reported in the singing group but not the film group.

**Conclusion:**

Singing classes have an impact on health status distinct from that achieved simply by taking part in a group activity.

**Trials registration:**

Registration Current Controlled Trials - ISRCTN17544114

## Background

Despite optimal treatment with pharmacological agents and pulmonary rehabilitation, many patients with chronic obstructive pulmonary disease (COPD) continue to be symptomatic [1,2]. A range of additional strategies to combat breathlessness have been trialed in respiratory patients [3], including pursed lip breathing [4,5], yoga and Tai Chi [6-9] and laughter [10]. There is evidence that singing can have beneficial effects on wellbeing in healthy [11] and chronic disease populations [12,13]. Since singing requires the use and mastery of techniques to control breathing, it is a plausible therapy for patients with respiratory disease and on the basis of a small number of clinical trials [14-16] the recently published IMPRESS British Thoracic Society Guidelines for Pulmonary Rehabilitation, have suggested that singing can be considered as an adjunct to this therapy [17].

We recently reported results from a pilot randomized controlled trial of singing classes for patients with COPD [15]. This showed that singing improved quality of life and anxiety but did not improve control of breathing measures, or functional exercise capacity. Patients who had participated in the trial reported benefits in their physical performance and general well-being as well as a sense of achievement and self-efficacy. Participants in an open program of singing classes were also overwhelmingly positive about the experience.

Based on these initial findings, we modified the protocol in several ways. Firstly, the control group had received “usual care” so it was possible that the benefits experienced were due to a non-specific effect of social interaction. Social isolation is an important issue for older people in general [18] and patients with COPD specifically [19]. We therefore incorporated an active control arm where participants took part in a film group. Secondly, the singing teacher had reported that she felt that after six weeks she was “only just starting to make progress” with many of the participants. Feedback from participants supported this, so the trial duration was increased to eight weeks. Thirdly, although the initial trial found no change in functional exercise capacity, assessed using the incremental shuttle walk test, patients reported that they felt able to do more and cope better with daily tasks, so we added physical activity monitoring to see if this translated into changes in daily activity. Finally, given the results of our initial trial the primary outcome was health status rather than the control of breathing measures adopted in the initial study.

The primary hypothesis of the present study was therefore that singing lessons would lead to a greater improvement in health status than a film studies group. In addition to health status, measures of exercise capacity, physical activity and breathing control were also assessed.

## Methods

Patients with COPD, diagnosed according to the GOLD guidelines, who were attending respiratory clinics at Royal Brompton and Harefield NHS Foundation Trust, were invited to participate. The trial was approved by the Brompton, Harefield and NHLI Research Ethics Committee. The trial procedures were explained to potential participants and all who took part in the trial gave written informed consent.

### Baseline assessments

Participants completed the Hospital Anxiety and Depression Scale (HADS) [20], COPD assessment test (CAT) score [21], and the Short Form 36 (SF-36) questionnaire [22]. Functional exercise capacity was assessed using the incremental shuttle walk test (ISWT) with time to recovery of oxygen saturation, Borg dyspnoea score and heart rate following the walk also documented [23].

Control of breathing was assessed using two measures in routine use in the physiotherapy department for the assessment of hyperventilation; a breath hold test where subjects held their breath from maximum inspiration [24,25] and single breath counting where subjects were instructed to breathe in and then count out loud in time with a metronome running at 60 beats/min [16]. The mean of three satisfactory attempts at each manoeuvre was recorded.

Each subject was then given a SenseWear Pro (SenseWear, Body Media, Pittsburgh, USA) activity monitor wear for one week prior to commencing their sessions and given written instructions on its usage and cleaning.

### Interventions

All subjects received a thirty minute standard session on breathing control and techniques to manage breathlessness, delivered by one of three senior respiratory physiotherapists involved in the study. Pursed lip breathing and nose breathing were also discussed in relation to managing episodes of shortness of breath. Each subject received a standard Royal Brompton Hospital “Help Yourself - physiotherapy for people with respiratory symptoms” booklet [15] and was advised to practice the techniques at home. At the end of the baseline visit, patients were randomized to either the singing classes or the film workshops, using randomization in blocks of 4 through consecutive sequentially numbered sealed envelopes. The sequence was developed by NSH who was not involved with the day to day conduct of the trial. Patients in both groups continued on their usual medications throughout.

The singing classes were held twice weekly for eight weeks and led by one of three singing teachers (MW, JS, PC). Each session lasted for one hour, and encompassed vocal exercises, posture and relaxation. The conduct of the singing groups is described elsewhere [15]. Subjects allocated to this group were given a CD of physical warm-ups, breathing exercises and songs to practice at home daily.

The film workshops were held once weekly and coordinated by a film-studies graduate (CW). Subjects watched the film together and then discussed any salient points in the workshop afterwards for about an hour. Films watched were as follows: Hable Con Ella, Local Hero, Vicky Cristina Barcelona, Point Blank, Punch Drunk Love, The Fog of War, Fargo, The Truman Show, Sunset Boulevard, Goodbye Lenin, Pan's Labyrinth, Double Indemnity, Shadow of a Doubt, The L Shaped Room, I Heart Huckabees, Little Miss Sunshine, The Fall, Vertigo.

The co-coordinators of each session were unaware of the tests measured at baseline.

Following attendance to either group for eight weeks, baseline measurements were again assessed by the same respiratory physiotherapists, who were blinded to treatment allocation. Subjects were asked not to discuss which group they had been allocated to. Study participants again wore the physical activity monitors for a further week and these were to be returned to the hospital on completion.

Statistical analysis was performed using StatView 5.0. Change in quantitative outcome measures between both groups was assessed using ANCOVA with baseline values included as covariates. Primary outcome was health related quality of life assessed using the SF-36. Based on our initial pilot data [15] with a standard deviation of 14.6 a 10 point difference in ΔSF-36 between groups would require a sample size of 20 to have a 90% power assuming a 2 tailed test and a significance level of <0.05.

### Qualitative assessments

Following completion of the randomized control study, a clinical psychologist interviewed a sample of subjects from each group. The structured interviews lasted for thirty minutes and used the same template as in our previous study [15]. Subjects were asked to discuss their perception of any physical and emotional benefits or harm they had experienced from attending the sessions. They were assured that their responses would be anonymous and that they could make negative comments if they wished.

## Results

### Comparison of response in singing and film groups

**Figure 1:**
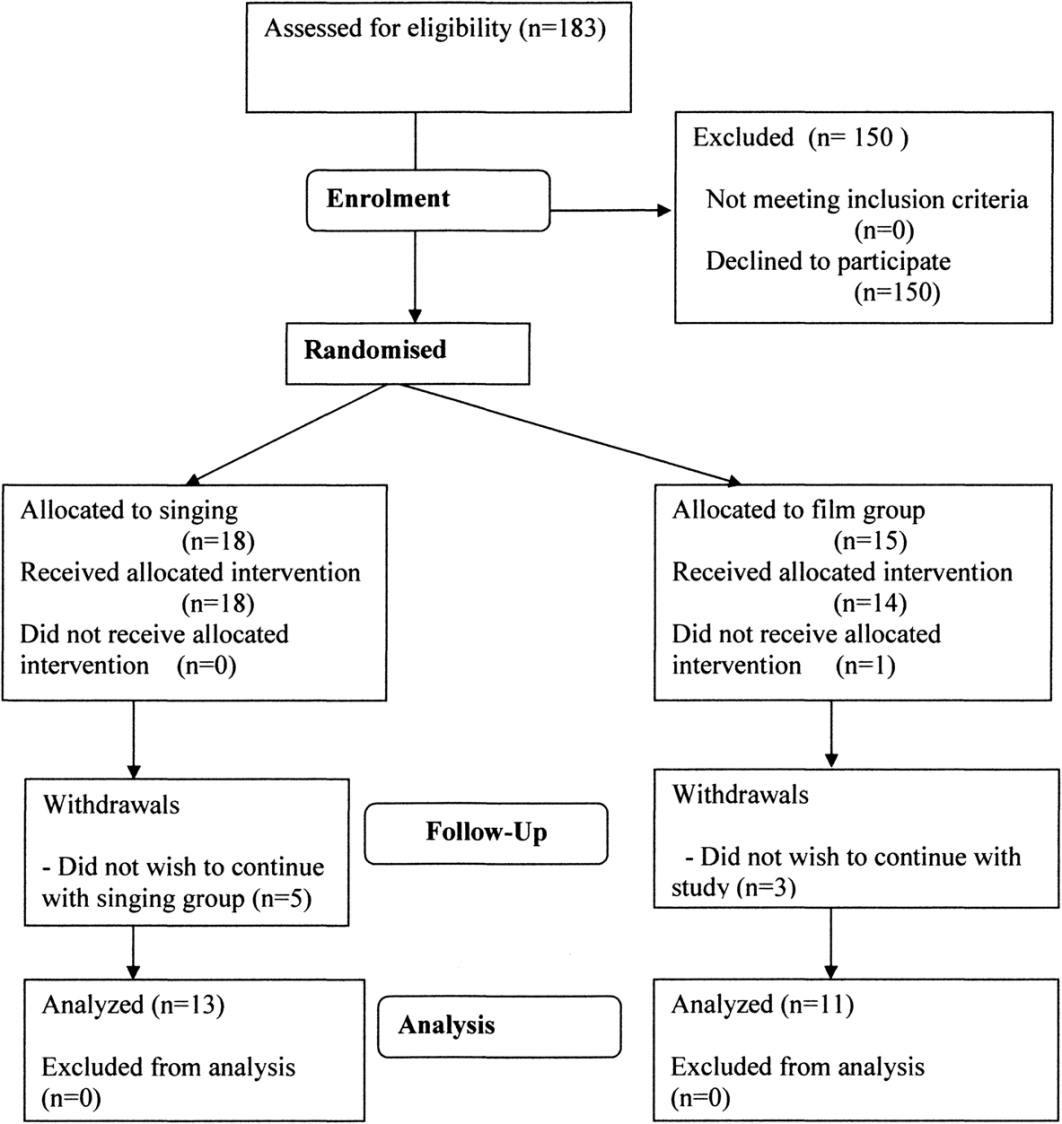
CONSORT diagram describing progress of the study.

The study took place between April 2010 and February 2011. Participation in the study is described in the CONSORT diagram (Figure 1). One hundred and eighty-three patients were approached to participate. One hundred and fifty declined to take part. Eighteen patients were allocated to the singing group and fifteen to the film one. Of the singing group, five subjects withdrew once the sessions had begun. In the film group, one subject died after randomization but before attending any sessions and three subjects withdrew from the study once the sessions had begun. Data are therefore presented on the thirteen subjects in the singing group and eleven in the film group who completed the study. Baseline characteristics of the two groups did not differ significantly (Table 1). Median number of sessions attended was 14.5 in the singing group and 7 in the film group.

**Table 1:** Baseline characteristics

	**Total (n=24)**	**Singing group (n=13)**	**Control group (n=11)**
Age (yrs)	68.3 (9.7)	68.6 (10.7)	67.9 (8.8)
FEV_1_% predicted	53.1 (22.0)	44.4 (14.4)	63.5 (25.5)
Exacerbation rate yr^-1^	1.83 (2.1)	2.00 (2.6)	1.64 (1.4)
Breath Hold (s)	27.8 (12.2)	29.0 (13.8)	26.3 (10.6)
Single Breath Counting (n)	19.8 (9.0)	21.5 (9.8)	17.9 (8.1)
HAD anxiety	5.58 (3.3)	5.69 (3.3)	5.45 (3.6)
HAD depression	6.00 (3.6)	7.23 (3.6)	4.5 (3.3)
SF36 PCS	42.8 (18.2)	36.5 (13.8)	50.3 (20.6)
SF36 MCS	53.6 (20.9)	48.2 (20.8)	60.0 (20.1)
CAT score	18.9 (8.86)	19.0 (10.03)	19.0 (7.7)
ISWT (m)	321.7 (168.9)	275.4 (196.1)	376.4 (115.6)
O_2_ sat^n^ recovery (s)	35.1 (44.8)	43.2 (5)	95.4 (50.2)
HR recovery (s)	147.5 (82.8)	144.4 (89.6)	25.5 (30.3)
Subjective recovery (s)	92.6 (47.8)	90.3 (47.6)	151.1 (78.1)
Steps (steps per day)	5791 (3463)	5277(3794)	6525 (3051)
Sedentary time (minutes per day)	856 (358)	934(262)	744 (463)
Physical activity duration (minutes per day)	457(263)	535(309)	402 (226)
Active energy expenditure (KJ per day)	1098(602)	1009 (506)	1223 (742)

FEV_1_ forced expiratory volume in one second; CAT, COPD assessment test score; HAD, hospital anxiety and depression score; SF-36 short form 36 health status score; PCS, physical component score; MCS, mental component score; ISWT incremental shuttle walk test; HR, heart rate (all p>0.05).

There were no significant differences between groups in the response of measures of breathing control, functional exercise capacity or daily physical activity (Table 2). However, although there were similar improvements in the mental component score of the SF-36 in both groups; Singing +9.3(25.3) vs Film +4.3(9.0) (p=0.41) there was a significant difference between the response of the physical component score favouring the singing group +12.9(19.0) vs -0.25(11.9) (p=0.02).

**Table 2:** Response to intervention

	**Singing group (n=13)**	**Control group (n=11)**	**p value**
Breath hold time (s)	−1.64 (4.1)	2.39 (7.8)	0.14
Single breath counting	1.5 (7.1)	7.0 (7.8)	0.15
HAD anxiety	−0.8 (3.6)	−0.9 (2.3)	0.89
HAD depression	−1.3 (3.8)	−0.7 (1.6)	0.49
CAT	−1.1 (8.3)	0.7 (5.6)	0.44
SF36 PCS	12.9 (19.0)	−2.5 (11.9)	0.02*
SF36 MCS	9.3 (25.3)	4.3 (9.0)	0.41
ISWT (m)	−7.2 (46.1)	14.5 (38.0)	0.22
Steps (steps per day)	−763 (1647)	1011 (1003)	0.14
Sedentary time (minutes per day)	−35.9 (127.3)	−27.3 (67.0)	0.66
Physical activity duration (PAD) (minutes per day)	−92.7 (216.9)	49.5 (40.9)	0.16
Active energy expenditure (AEE) (KJ per day)	−144.2 (436.0)	228.8 (146.3)	0.19

FEV_1_ forced expiratory volume in one second; CAT, COPD assessment test score; HAD, hospital anxiety and depression score; SF-36 short form 36 health status score; PCS, physical component score; MCS, mental component score; ISWT incremental shuttle walk test; HR, heart rate. (*p<0.05).

### Qualitative survey of patient experiences

Nine subjects who had taken part in the study were interviewed: five singers and four from the film group. One interview was conducted via telephone; the remaining eight interviews took place at the Hospital. As in our previous study, these are presented in terms of physical and general wellbeing, with the latter divided into sub-categories.

All participants in the singing group reported positive physical effects in relation to their breathing following attendance at the singing group. In particular, they reported being more aware of their breathing and how to control it more effectively. Comments included: *“Helped my fitness…using the breathing technique in the gym and everyday life” “Have more control over my breathing…know how to use my breath more” “Group was good…bit like a Pilates workout but better than going to the gym which is boring” “Feel I’m doing a bit more light housework, like sweeping and laundry” “Learnt something about breathing through the singing and about pacing”.* Two participants reported multiple physical health conditions. They both reported that they felt that it had been hard to assess any physical benefits due to their multiple conditions.

In general, film group participants did not feel they received any positive physical effects from attending the sessions. One participant felt that attendance at the film group had been good for his health *“in terms of enjoyment”.*

The singing group participants reported multiple benefits in terms of their general wellbeing. Relating to mood and pleasure, participants reported: *“Helped with my mood”, “Nice to be doing something pleasant with a chronic illness” “Great fun” “Enjoyed talking to others about singing” “Depressing being in all the time…group got me out of the house” “Immensely enjoyed it” “Positive impact on my mood…we’re singing in the pub tonight for the open mic”.*

The singing group participants reported that they had experienced a sense of community and social support: *“Being around others with COPD prepares me for the future…helps me to learn more about my illness” “Others understood what I was saying about my chest…gave me tips to talk to my doctor about” “Meeting other people with similar illness helped…felt like everyone understood me…didn’t look down on me like at the gym” “Peer support…people the same as you are!” “Nobody was judgmental!” “Psychological benefit of seeing people”.*

In terms of achievement and efficacy, singers reported: *“Want to build singing into daily routine…helps me to overcome difficulties” “Hadn’t felt like going out before course due to breathing problems…now feel I can overcome anything” “Had forgotten how much I enjoyed singing” “Singing group got me out of the house”.*

A singing group facilitator commented that sixteen sessions seemed about the right duration but that mixed ability classes were challenging and that this could be more difficult with a rolling program where new people were joining the group.

Participants in the film group did report some positive effects on their general well-being. In terms of mood and pleasure: *“Enjoyed movies and enjoyed learning from group leader” “Enjoyed it…I don’t relax a lot”* One participant reported negative effects the film group had had on her general well-being: *“Increased my anxiety…I’m a very religious person…lots of films I wouldn’t choose to see…relieved it’s over”.* In the film group participants did experience a sense of community and social support: *“Like a club day…mixing and meeting different people” “Good discussions” “Sense of social support” “Felt supported by peers in group”.* One participant reported the film group was not large enough to provide social support. In terms of achievement and efficacy: *“Group made me get out of the house which is important” “Made me think about things differently” “New experience…wouldn’t have done it normally.*”

The film group participants felt that there had been little benefit in attending the group but that it had not caused them any significant harm either. All the film group participants reported that they would like to enrol in the singing classes due to the benefit they perceived would come from that approach.

The findings highlighted how enjoyable the participants found the singing group. As well as providing social support, participants felt that the group had had lasting positive physical effects in terms of their breathing techniques. All the participants were keen to continue to use what they had learned within the group. Participants felt that they had achieved something both personally and physically.

In comparison, the participants from the film group did not report any significant positive physical effects from their group attendance although they had in general enjoyed the experience and felt they had gained some social support from attending. Part of the process of the film club sessions involved considerable discussion amongst the subjects on topics raised by the film they had just seen, and on the films that they had been inspired to see in their own time. These discussions could last up to an hour and increased over the weeks the film club sessions ran. The facilitator reported that *“It did seem to me that talking this much was often an effort for them but they were motivated to carry on. It also seemed to me that there was initially insecurity towards involvement with the group discussions, but this slowly dissolved*”.

## Discussion

The main findings of this study are that 1) the singing classes were associated with an improvement in the physical component score of the SF-36 compared to the film group which exceeded the minimum clinically important difference for this test [26]. 2) Qualitative data from interviews with participants highlighted perceived physical and psychological benefits from attending singing rather than the film group although experiences of the latter were generally positive. 3) The difference in perceived improvements between the two groups was not accompanied by differences in measures of breathing control, functional exercise capacity or daily physical activity.

### Significance of findings

The present data develop the findings of our previous work in this area which showed improvements in health status and a positive patient experience associated with participation in singing classes [15]. In the previous study the control group received only usual care so it was possible that the reported benefits were due to a reduction in social isolation. A strength of the present study is that there was an active control group who took part in a group activity which means that some of the “generic” effects of group activity on social isolation can be discounted. Thus although the film group reported that the experience was generally positive they did not report improvements in their physical health in contrast to the singing group.

In addition to our previous study [15] we are aware of one other controlled trial of singing in patients with COPD. In that study patients either attended singing classes, or a handicraft class once a week for 6 months [14]. Although singing practice produced an acute improvement in inspiratory capacity, SGRQ improved equally in both groups. Exercise capacity was not assessed.

Patients with COPD have increased operating volumes, increased neural drive and display changes in the excitability of corticospinal pathways to the diaphragm [27-30] meaning that there are a range of mechanisms through which relief of breathlessness could occur. The improvement in health status may have arisen directly through effects on breathing pattern, posture or the learning of techniques to hasten recovery from episodes of dyspnoea but we have no specific data on physiological changes to address these questions. Interestingly, these perceived improvements were not reflected in objective outcome measures. This was a relatively small pilot study but it does raise interesting questions about the appropriateness of outcome measures for this sort of complex and relatively personalized intervention. Although they were not significantly different, the control of breathing measures, as in our previous pilot, paradoxically favored the non-singing group. These tests were developed for evaluating hyperventilation so may not be appropriate for COPD patients. As both relate to breath-holding it may be that participants in the singing group had learned to make a more relaxed and thus less deep inhalation leading to worse performance on these specific tests.

Physical activity limitation is an important feature of COPD being associated with muscle weakness, more rapid disease progression and reduced health status [31-33] so the absence of improvement in measures of physical activity was disappointing but may have been due to the small sample size, as responses were highly variable.

Many patients approached did not wish to participate. A range of reasons were cited, many logistical, including the time commitment and believing that singing would be helpful so not therefore wishing to be randomized to the non-singing control arm. Of note, all of the film group participants wanted to go on to take part in singing classes. Translating this into clinical practice, singing is more likely to be of benefit to patients who believe it will be helpful. The present data suggest that singing does produce specific benefits and that participation in singing classes should be encouraged where these are available. It is certainly not a substitute for a proper multidisciplinary pulmonary rehabilitation program [2,34] and further work is needed to see if it can be applied directly as an adjunct during pulmonary rehabilitation or perhaps as an element of a continuation program.

A further practical issue is the duration of the intervention. Different models are possible ranging from an intensive one to one approach continued indefinitely through a fixed period of sessions through to a long term group approach with flexible participation. Decisions about this are likely to be dependent on practical and economic factors. A rolling rather than a fixed program may offer greater flexibility for participants but has the disadvantage that this may impede the group’s progression as they acquire new skills because “beginners” will need to be taught basic techniques. It is also worth noting that over half the session-time was normally spent on physical warm-ups, breathing exercises and singing exercises; under half singing songs: this proportion is perhaps different to a ‘normal’ community singing group in its emphasis on technique over song. A further issue is group size. Sessions usually involved 4 to 6 people and this would be a different experience to taking part in a larger choir an issue that could be addressed in future work.

A limitation of the study is that the singing group was twice weekly and the film discussion weekly which may have influenced the “dose” of social interaction with the singing group spending more time in the group activity. However, the qualitative data suggest that both interventions had social effects of a similar nature but that participants in the film group experienced no “physical” effect, making it unlikely that there would have been a different outcome if the film group had met more frequently.

## Conclusion

The present data suggest that singing has specific effects of physical wellbeing and taken together with other small studies support the concept that participation in singing lessons may be a useful activity for patients with COPD.

## Competing interests

The authors declare they have no competing interests.

## Authors’ contributions

NSH, VML, VJH, PC, JLK and MIP conceived the study; VML, CS, MS, RT and JLK performed the study measurements. PC, JS, PC and CW delivered the study interventions. VML, VJH, AE and NSH performed the data analysis. VML and NSH wrote the first draft of the paper to which all authors subsequently made contributions. All authors read and approved the final manuscript.

## Pre-publication history

The pre-publication history for this paper can be accessed here:

http://www.biomedcentral.com/1471-2466/12/69/prepub
