# Erratum to: Singing classes for chronic obstructive pulmonary disease: a randomized controlled trial

**DOI:** 10.1186/1471-2466-14-181

**Published:** 2014-11-19

**Authors:** Victoria M Lord, Victoria J Hume, Julia L Kelly, Phoene Cave, Judith Silver, Maya Waldman, Chris White, Cayley Smith, Rebecca Tanner, Melissa Sanchez, William D-C Man, Michael I Polkey, Nicholas S Hopkinson

**Affiliations:** NIHR Respiratory Biomedical Research Unit at Royal Brompton and Harefield NHS Foundation Trust and Imperial College, Sydney Street, London, SW3 6NP UK

In our 2012 paper on the effect of singing classes for patients with COPD [1] which compared them to participation in a films studies group, we cited the wrong clinical trial number – (ISRCTN17544114). This number in fact relates to a previous study we had undertaken, published in 2010 which also evaluated singing classes in COPD but had compared them to a “usual care” arm [2].

The trial number we should have cited is ISRCTN39714922.
